# Reduction of gene repair by selenomethionine with the use of single-stranded oligonucleotides

**DOI:** 10.1186/1471-2199-8-7

**Published:** 2007-01-26

**Authors:** Timothy R Schwartz, Eric B Kmiec

**Affiliations:** 1 Department of Biological Sciences, University of Delaware, Delaware Biotechnology Institute, 15 Innovation Way Newark, DE 19711, USA

## Abstract

**Background:**

The repair of single base mutations in mammalian genes can be directed by single-stranded oligonucleotides in a process known as targeted gene repair. The mechanism of this reaction is currently being elucidated but likely involves a pairing step in which the oligonucleotide align in homologous register with its target sequence and a correction step in which the mutant base is replaced by endogenous repair pathways. This process is regulated by the activity of various factors and proteins that either elevate or depress the frequency at which gene repair takes place.

**Results:**

In this report, we find that addition of selenomethionine reduces gene repair frequency in a dose-dependent fashion. A correlation between gene repair and altered cell cycle progression is observed. We also find that selenium induces expression of Ref-1 which, in turn, modifies the activity of p53 during the cell cycle.

**Conclusion:**

We can conclude from the results that the suppression of gene repair by introduction of selenomethionine occurs through a p53-associated pathway. This result indicates that the successful application of gene repair for treatment of inherited disorders may be hampered by indirect activation of endogenous suppressor functions.

## Background

Targeted gene repair is a process that corrects single base mutations in genes within the chromosome. Nucleotide alteration is directed by a short single-stranded oligonucleotide, which also serves as a template for the base exchange reaction. The mechanism of action continues to be unraveled but the general system involves a DNA pairing step in which the oligonucleotide aligns to the homologous target site and a correction step in which the genetic information in the oligonucleotide is transferred to the target gene [1,2]. The reaction is catalyzed by enzymes involved in homologous recombination and may be influenced by proteins that regulate the cellular response to DNA damage [3-6]. Damage occurs naturally in mammalian genomes by the action of oxygen radicals, mistakes in DNA synthesis and from exogenous sources such as chemicals or irradiation.

Chemotherapeutic drugs such as VP16 or camptothecin (CPT) or reagents such as hydroxyurea, thymidine, or methyl methanesulfonate (MMS) that cause lesions to accumulate at replication forks [7-10] can be used to induce DNA damage in experimental systems and facilitate studies of gene repair. These lesions inhibit fork movement, leading to a stall in S phase, an event that stimulates gene repair activity. In a recent study, Hu *et al*. [11] found that the synchronization of cells at the G1/S border with subsequent release enables a higher level of gene repair. The correction levels peak when the majority of cells are in S phase, results which align with earlier data of Majumdar *et al*. [12]. In addition, Brachman and Kmiec [13,14] demonstrated that DNA replication can influence both the rate and frequency of gene repair. By slowing fork movement, these workers were able to elevate the frequency by activating the homologous recombination (HR) pathway and enabling the correction process to occur with greater efficiency. These data are consistent with earlier reports that had already pointed to HR as a regulatory process of gene repair [4,10]. The induction of HR pathways by DNA damage and the subsequent stimulation of gene repair has provided further clarification of the proteins and pathways involved in the correction events. While earlier findings support a role for DNA damage-induced responses in elevating the frequency of gene repair, little is known about the mechanism of correction *i.e*. the details of nucleotide exchange. What is known is that regulatory proteins activated by DNA damage events stimulate a cellular response that leads directly or indirectly to a high efficiency of gene repair activity.

The p53 tumor suppressor regulates cell cycle checkpoints, apoptosis, and can transactivate a number of genes involved DNA repair. Furthermore, p53 can act to suppress HR; it is recruited to stalled replication forks to slow or prevent uncontrolled homologous exchanges [15-17]. When p53 was overexpressed in DLD-1 cells simultaneous with the initiation of oligonucleotide-directed gene repair, p53 acts as an antagonist of the reaction, reducing the level of eGFP positive, corrected cells significantly [13]. This suppressive activity could result from p53's role in cell cycle control, DNA damage response, or regulation of senescence and apoptosis. As a long term goal, we seek to modulate and harness the HR repair response as it relates to gene repair activity without inducing the natural suppression associated with the induction of DNA damage.

The amino acid selenomethionine, the major component of Se-enriched foods, has long been demonstrated as a chemopreventative agent, and selenium compounds have yielded encouraging results in current clinical trials to prevent prostate, colon, and other human cancers. Selenomethionine has been reported to induce a DNA repair response and enhance repair-complex formation in treated cells by means of a modulation of p53 activity [18]. The tumor-suppressor function of p53 is usually attributed to its activity at a post-damage stage when it eliminates cells with damaged DNA resulting from genotoxic stress. Selenomethionine seems to induce a different branch of p53 activity, converting p53 into a new conformation through a reduction reaction catalyzed by the protein Ref-1. In this form, p53 does not have any growth-suppressing effect but can effectively modulate DNA repair. Thus, p53 facilitates control over genomic stability by a mechanism that does not require cell death or arrest [18,19]. An agent that modulates gene repair without exhibiting toxicity or inducing DNA damage has numerous advantages, particularly in cases where the level of repair falls below that of clinical relevance and an adjuvant treatment will be required.

Ref-1 is important for the redox regulation of p53, but it is also involved in a number of critical cellular pathways. Ref-1 associates with the human Ape1 protein, forming the multifunctional human AP endonuclease (Ape1/Ref-1). Ape1-Ref-1 functions as an AP endonuclease in DNA repair, and it can activate numerous transcription factors through redox-dependent and redox independent mechanisms [20]. Ref-1 exerts redox control of p53 protein by reducing adjacent cysteine residues (at position 275 and 277 respectively), breaking the disulfide bond and rendering a new p53 conformation. This conformation is associated with increased activation of DNA repair proteins, thus requiring less action for cell cycle arrest or apoptosis.

As described above, selenomethionine has been reported to induce a DNA repair response and enhance repair-complex formation in treated cells. We have reported how to create such a response by inducing a tolerable level of DNA damage that elevates gene repair activity [5,6,21]. In order to advance the technology of gene repair in disease models it is necessary to develop safe and simple methods to increase the efficiency of this repair. Selenium compounds clearly influence DNA damage response pathways [22] often modulating the levels of enzymes known to be involved in gene repair. It should be noted that these authors focused on nucleotide excision repair, a process unrelated to homologous recombination. In any event, we examined the effect of selenium on gene repair activity in a mammalian model cell system in order to gain a greater perspective on the degree of cellular response to the activation of repair pathways.

## Results

### The assay system

**Figure 1:**
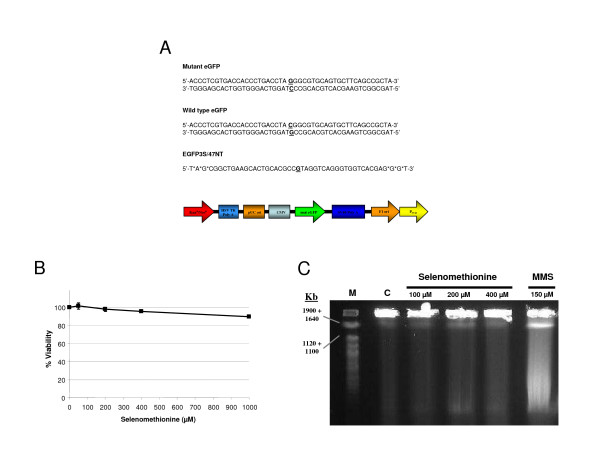
***(A). ****Map of mutant eGFP gene, integrated in HCT 116 cells*. The wild-type and mutant sequence of the eGFP gene, where the point mutation is in the eGFP cassette, TA*G*, are shown above (base is in bold and underlined). This is the target for the 47-mer oligonucleotide, EGFP3S/47NT, directed to the non-transcribed strand of the eGFP gene. EGFP3S/47NT is a single-stranded oligonucleotide with three phosphorothioate modifications on each terminus. **(*B)****. Selenomethionine is not cytotoxic in HCT 116 cells*. Cells were incubated with selenomethionine at concentrations of 0, 50, 200, 400, and 1000 μM, respectively. Viability was determined by an MTT-reduction assay; each data point represent mean of three (± S.D.), non-treated cells are normalized to 100% viable. **(*C)****. Exposure to selenomethionine does not induce DSBs in HCT 116 cells*. Cells were grown with respective treatment for 24 hours prior to harvesting and prepared for pulsed-field gel electrophoresis (PFGE). The gel was run for 24 hrs and subsequently stained with ethidium bromide. Lane M, *S. cerevisiae *chromosomal DNA size marker presented in kilobases; Lane C, non-treated control HCT 116 genomic DNA; Selenmethionine-treated (100 μM, 200 μM, 400 μM) HCT 116 cells; Lane MMS, MMS (150 μM) -treated HCT 116 genomic DNA.

A standard assay system based on the correction of an integrated mutant eGFP gene was employed to measure gene repair activity. A single clonal isolate from HCT 116 cells (which contain the wild-type complement of p53 genes) containing a single copy of the mutant eGFP gene (which is actively transcribed in these cells) was used for these experiments. A low-copy target gene is advantageous in a model system for oligonucleotide-directed gene repair, conferring only a single site for gene repair activity per cell. Specific oligonucleotides were designed to correct the TA G point mutation at amino acid position 67 which lies in the chromophore region of the eGFP gene and conversion to TA C enables expression of wild-type eGFP, allowing detection of positive conversion events by FACS analysis [11] (see Figure 1A). The standard vector, EGFP3S/47NT, is a 47-mer containing three phosphorothioates at each termini to protect against nuclease activity. This system is well established and has been used in multiple studies to validate gene repair activity [5,6,11,13,23].

### Selenomethionine does not induce cell toxicity

We examined cellular toxicity and genotoxicity of selenium in the HCT 116 model system. In general, organic selenium compounds have been found to exhibit low levels of toxicity in most cells. In our hands, selenomethionine was found to be nontoxic in HCT 116 cells, as measured by an MTT reduction assay for viability with concentrations up to 1 mM (see Figure 1B). These results set the viability parameters and indicate that a 24 h pretreatment with 50–200 μM selenomethionine, the experimental conditions used traditionally to assess the modulation of gene repair activity, does not lead to significant cell death.

### Selenomethionine does not induce double-stranded breaks in DNA

At the genomic level, no significant DNA breakage in HCT 116 cells is observed by the addition of selenomethionine as evidenced by the pulse field gel (PFGE) presented in Figure 1C. Genomic DNA isolated from selenomethionine-treated cells at concentrations of 100, 200, and 400 μM respectively does not exhibit greater fragmentation compared to an untreated population of cells. This is in sharp contrast to the positive control in which the cells were treated with methyl methanesulfonate (150 μM) [24]. In this case, significant fragmentation is observed, evidenced by the bright smear of DNA containing an extensive number of breaks running into the gel. The bright band near the top of the damaged DNA region represents genomic DNA containing only a few strand breaks. Thus, selenomethionine treatment does not result in extensive DNA damage and therefore does not induce a cellular DNA repair response similar to that seen when etoposide (VP16) or camptothecin is added to a cell culture [25].

### Gene repair activity modulated by selenomethionine

**Figure 2:**
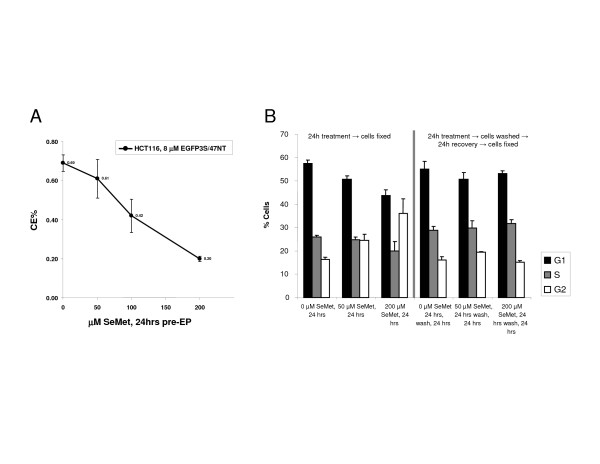
***(A)****. Selenomethionine treatment suppresses gene repair*. HCT 116 cells were treated with selenomethionine (50 μM, 100 μM, 200 μM) for 24 hours prior to electroporation of the oligonucleotide. The cells were analyzed by FACS 24 hours after electroporation. The data represents three (± S.D.) independent experiments. **(*B)****. Selenomethionine induces mild, reversible G2/M delay*. HCT 116 cells were not treated or treated with selenomethionine (50 μM, 200 μM) for 24 hours and processed for analysis of cell cycle. The profiles were generated using ModFit LT software. For cells treated with selenomethionine for 24 hrs: no treatment (57.6 +/- 1.4%G1, 26.1 +/- 0.6%S,16.37 +/- 0.8% G2), 50 μM selenomethionine (50.7 +/- 1.6% G1,24.7 +/- 1.3%S,24.6 +/- 2.6% G2) 200 μM selenomethionine (43.8 +/- 2.27% G1,20.1 +/- 3.9% S,36.1 +/- 6.2 % G2). For cells treated with selenomethionine for 24 hrs, washed, and allowed to recover for 24 hrs: no treatment (55.2 +/- 3.3% G1,28.8+/- 1.8S,16.1 +/- 1.4%G2), 50 μM selenomethionine (50.8 +/- 2.9% G1, 29.8+/- 3.3% S,19.4+/- 0.4%G2) 200 μM selenomethionine (53.2 +/- 1.2% G1, 31.7+/- 1.9% S,15.1+/- 0.7 G2).

Recently, we reported that inducing a low level of DNA damage in cells significantly elevates the level of gene repair activity [5,6]; the introduction of double strand breaks in the target cell led to a cell cycle arrest in response to the creation and/or presence of the DNA damage. During the arrested phase of the reaction, the oligonucleotide is able to more accurately and efficiently gain access to the target site on the DNA, presumably because the cells undergoing gene repair are contained within the population suspended in S phase [see 26 for review]. One response to DNA damage is the elevation of activities which have been shown previously to regulate the gene repair reaction. Thus, agents that increase repair activity are obvious candidates for stimulatory factors of correction, and particularly those that stimulate the repair pathways without causing DNA damage. Along these lines, selenomethionine has been shown to induce DNA repair processes by stabilizing the formation of repair complexes [27]. Thus, we tested for modulation of gene repair by pre-treating the cells with varying doses of selenomethionine. The cells were treated for twenty-four hours, followed first by a wash-out and electroporation of the oligonucleotide, EGFP3S/47NT. Gene repair was evaluated by FACS twenty-four hours later, and the data reveal that correction levels decrease as a function of dose of selenomethionine (50, 100, and 200 μM, respectively) (Figure 2A). These are the same concentrations (described above) that exhibit no significant cytotoxicity (see Figure 1B).

### The effect of selenomethionine treatment on cell cycle progression

As mentioned above, some treatments that increase the efficiency of gene correction do so by inducing DNA damage and cell cycle arrest [26]. Other treatments induce S phase accumulation but without DNA damage and still support high levels of gene repair [13,14]. Since selenomethionine has not been reported to damage DNA directly and we observed no ds breaks (see Figure 1C), we examined the effect of selenomethionine pretreatment on cell cycle progression. As shown in Figure 2B, cells treated with selenomethionine for 24 hrs exhibit a different cell cycle profile compared to untreated cells. FACS analysis reveals that while the overall percentage of cells in S phase is the same for non-treated and selenomethionine-treated cells, selenomethionine-treated cells exhibit some accumulation in G2 with a compensatory reduction in the population of cells in G1 or at the G1/S border. This result is similar to a cell cycle block caused by oxidative damage, where the passage from G2 to mitosis is delayed [28]. Thus, selenomethionine does not arrest or delay cells in S phase, a condition that has been shown to stimulate gene repair activity. Interestingly, the effect on cell cycle is reversible, as the cell cycle profile of treated cells returns to normal 24 hours after the removal of selenomethionine from the culture (Figure 2B).

### Selenomethionine does not lead to increased expression or activation of p53 but does increase Ref-1 levels

**Figure 3:**
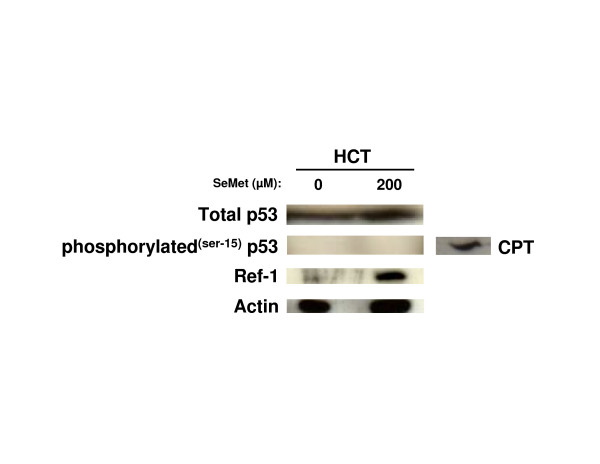
*Selenomethionine treatment does not result in great levels of expressed or activated p53*. HCT 116 cells were not treated or treated with selenomethionine (200 μM) for 24 hours prior to protein isolation. Western blot analysis reveals that expression of wild-type p53 is unchanged in the presence of selenomethionine; activation of p53 by serine-15 phosphorylation does not occur in the presence of selenomethionine; p53 is phosphorylated at serine-15 when cells are treated with camptothecin (CPT; 30 nM) for 24 hours; it serves here as a positive control.

As shown in Figure 2A, selenium reduces gene repair activity. One may therefore predict that this reduction is transmitted through a Ref-1-mediated redox activation of wild-type p53 [18], which has been shown to suppress gene repair activity [13]. As such, selenomethionine treatment should not increase the overall expression of p53 in HCT116 cells, nor should it induce the activation of p53 through phosphorylation. We tested this prediction by western blot analysis of proteins isolated from HCT116 cells treated with selenomethionine (200 μM) for 24 hours (Figure 3). An immunoblot with the p53-DO antibody, which is specific for total wild-type and mutant p53, exhibited no distinguishable change in p53 expression in non-treated or selenomethionine-treated cells. Likewise, immunoblotting with an antibody specific to an activated form of p53 (phosphorylation of serine-15) [29,30], which occurs in response to DNA damage showed no detectable activated p53 in the non-treated and selenomethionine-treated cells.

Whereas p53 levels are unchanged in the presence of selenomethionine, the Ref-1 protein is present at higher levels in HCT116 after 24 hrs treatment with selenomethionine (200 μM), as determined by immunoblot specific to Ref-1 (Figure 3). This supports our hypothesis that selenomethionine may reduce gene repair activity through p53 modification that is mediated by the Ref-1 protein.

### Effect of p53 overexpression (wild-type and mutant) in human cell lines

**Figure 4:**
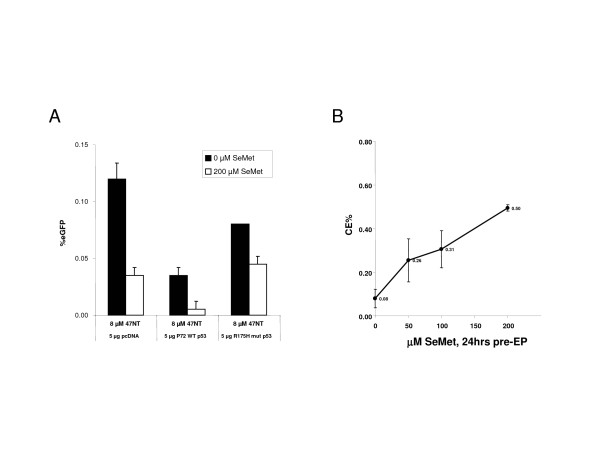
***(A)****. Suppression of gene repair by p53 overexpression is equivalent to selenomethionine treatment*. Cells were incubated with or without 200 μM selenomethionine for 24 hrs. 5 μg of respective overexpression plasmid (empty plasmid (pcDNA), wild-type p53 (P72), or mutant p53 (R175H)) and 8 μM EGFP3S/47NT oligonucleotide was electroporated in HCT 116 cells; gene repair levels were evaluated by FACS after 24 hours. **(*B)****. Selenomethionine treatment enhances gene repair in DLD-1 (p53+/- mutant)*. DLD-1 cells were treated with selenomethionine (50 μM, 100 μM, 200 μM) for 24 hours prior to electroporation of the oligonucleotide. The cells were analyzed by FACS 24 hours after electroporation. The data represents three (± S.D.) independent experiments.

Wild-type or mutant p53 overexpression plasmids were introduced to cells in the presence of selenomethionine with simultaneous introduction of the targeting oligonucleotide. 5 μg of respective p53 overexpression plasmid and EGFP3S/47NT oligonucleotide were electroporated and evaluated by FACS after 24 hours. As displayed in Figure 4A, cells transfected with the pcDNA empty plasmid and selenomethionine exhibit a reduction in gene repair activity. This result is equivalent to suppression of gene repair activity found in cells transfected with the p72 plasmid, which overexpresses wild-type p53. In combination, selenomethionine treatment in the presence of overexpressed p53 exacerbates this effect further, resulting in a nearly undetectable level of correction. Thus, selenomethionine-dependent redox control of endogenous p53 appears to act synergistically with elevated levels of p53 in the suppression of gene repair.

To explore the role of p53 in this reaction, we utilized a separate cell line that has been used previously as an assay system for gene repair activity. This cell line, DLD-1 [11] contains the same mutated eGFP gene as the HCT116 line used above in low copy number. Using a cell line with a low copy number of integrated targets is important in studies related to gene repair. In addition, this line does not have a full complement of p53 [see 14]. Using the same reaction protocol as carried out for experiments in HCT116 cells, the integrated DLD-1 cell line was electroporated with EGFP3S/47NT and the appearance of corrected cells viewed 24 hours later. Some samples were pre-incubated with the indicated concentration of selenomethionine for 24 hours prior to electroporation. As shown in Figure 4B, selenomethionine does not induce an inhibition of gene repair; in fact a slight but reproducible stimulation is seen in a dose-dependent fashion. Thus, a cell line devoid of the full complement of wild-type p53 responds differently to treatment with selenomethionine than does a cell line containing wild-type p53.

## Discussion and conclusion

The product of the tumor suppressor gene, p53, down-regulates the process of homologous recombination [17,31]. The protein may act upon enzymes involved in the DNA damage response pathway or act directly at crossover junctions, effectively stalling their development. Since the process of gene repair requires the activity of proteins involved in HR, we might predict that events that induce HR suppression would likewise result in a reduction in the frequency of gene repair. Previous work in our laboratory [13] and others demonstrated that wild-type p53 can act as an antagonist of the gene repair reaction, whereas overexpression of some mutant p53 proteins exhibit a small yet significant elevation in the frequency of gene repair [13]. The suppressive action of wild-type p53 [17] may affect the gene repair reaction through the binding to replication forks which have been shown to be a key intermediate (and in fact a stimulant) in the correction process [26].

We observe that the overexpression of wild-type p53 gene in HCT116 leads to a reduction in gene repair, confirming data from Pierce *et al*. [32] and Brachman *et al*. [13]. The study by Pierce *et al*. utilized cell-free extracts as sources of gene repair activity while Brachman and Kmiec tested p53 overexpression directly in cells [13]. The inhibitory effect of p53 in DLD-1 cells which contain both mutant and wild-type alleles was clearly evident. Mutations in the DNA binding domain of the p53 gene can cause the p53 protein to lose the ability to suppress homologous recombination [31,33,34]. Mutant forms of p53 may not be able to inhibit Rad51-mediated strand exchange or the reverse branch migration of stalled replication forks [17,35]. Expression of several mutant p53 proteins, specifically, the p53 175H mutant, which inhibits G1 checkpoint control, can prevent the suppression of HR and stimulate replication inhibition-induced HR [9,15]. Here, we utilize HCT116 cells that have a normal genetic complement of wild-type p53 genes and show that additional p53 suppresses the frequency of gene repair even further.

Our data suggest that suppression of gene repair activity most likely does not occur through an elevation in p53 expression nor by the activation of a well-known p53 conformer. Thus, we explored a novel explanation for the suppressive nature of selenomethionine in the gene repair reaction [20,36], showing that the addition of selenomethionine (SeMet) to HCT116 cells leads to a reduction in gene repair activity. Selenium compounds have been shown to elevate p53 activity [37,38] and to stimulate DNA damage response pathways through a phosphorylation cascade that includes ATM and H2AX [22]. Gene repair activity has been shown to be sensitive to the activities of proteins involved in the response to DNA damage [13,14]. Thus, it was of interest to determine which selenium-induced pathway would predominate in this system; would increased DNA repair activities promote correction or would induction of p53 lead to suppression. We observed that gene repair is possibly inhibited by a pathway involving p53. This pathway includes Ref-1 which controls the activity of p53 through redox activation [20]. Ref-1 is also a key protein in the activation of other proteins including transcription factors and enzymes involved in nucleotide excision repair [20]. While the downstream effects of Ref-1 induction are numerous, we focused on the activation of the Ref-1 protein by SeMet. We show that under conditions that promote gene repair, SeMet induces Ref-1 expression with a concurrent diminution of correction. The reduction by SeMet aligns with the effect of p53 overexpression (Figure 4A and 4B) and the two appear to act synergistically. Taken together, these data suggest that inhibition of gene repair by SeMet most likely takes place in a pathway involving Ref-1. The most likely downstream target for Ref-1, especially in response to DNA damage is p53. Thus, consistent with the previous reports, we believe that p53 plays a critical, if not suppressive role, in regulating the frequency of gene repair.

Our data are consistent with a role for homologous recombination in the mechanism of gene repair [26]. A number of key proteins are involved in modulating the frequency of correction. These include Rad51, Rad52 and Rad57 [39] as well as ATM, CHK1 and CHK2 [40]. Based on the data presented herein, we can conclusively add p53 to that list. This protein can inhibit homologous recombination functions by binding to the three-stranded recombination intermediate and destabilizing it [35,41]. As shown by Drury *et al*. [42], the three-stranded intermediate is a requisite structure in the gene repair pathway and thus one can envision that p53 suppression activity occurs through the destabilization of structural intermediates. Alternatively, the activation of p53 by selenomethionine, mediated by Ref-1, could inhibit the progression of replication forks, by enabling fork regression [17,43]. This reversal reduces the number of targets for gene repair because correction events rely heavily on the process of DNA replication [26]. Thus, induction of p53 activity will remain a barrier to the successful application of gene repair as therapy for genetic disorders.

## Methods

### Cell line and culture conditions

The HCT 116 cell line was acquired from ATCC (American Type Cell Culture, Manassas, VA). The HCT 116 was created by the integration of a pEGFP-N3 vector (Clontech, Palo Alto, CA) containing a mutated eGFP gene, as described by Hu *et al*. [11]. A nonsense mutation at position +67 results in nonfunctional eGFP protein; the oligonucleotide directs the conversion of the stop codon to a tyrosine (wild-type eGFP), allowing the expression of functional eGFP. HCT 116 cells were grown in McCoy's 5A Modified medium (Sigma-Aldrich, St. Louis, MO) and 10% fetal bovine serum. The mutant eGFP gene target was integrated into these cells and a clonal line containing a single copy was isolated by neo-selection (Geneticin, GIBCO, Invitrogen, Carlsbad, CA). DLD-1 integrated cell line was created and transfected as described by Hu *et al*. [11].

### Selenomethionine treatment

Cells were seeded at a density of 1.5 × 10^6 ^in a 100 mm dish in complete medium supplemented with 10% FBS and desired concentration of selenomethionine (Sigma-Aldrich, St. Louis, MO) 24 h prior to electroporation of the oligonucleotide. All cells were washed twice in 1X PBS (GIBCO, Invitrogen, Carlsbad, CA) immediately prior to harvesting for electroporation.

### MTT viability assay

Sensitivity to selenomethionine was analyzed by a 3- [4,5-dimethylthiazol-2-yl]-2,5-diphenyltetrazolium bromide (MTT, Sigma-Aldrich, St. Louis, MO) viability assay. Cells were plated in a 24-well plate at a density of 1 × 10^5 ^cells per well using complete medium supplemented with 10% FBS and selenomethionine at concentrations of 50, 200, 400, and 1000 μM, respectively. After 24 hours of treatment, cells were washed in PBS and 500 μl (5 mg/ml) MTT in RPMI was added to each well and incubated for 2 hours at 37°C, in the dark. This assay is based on a reaction in which MTT is reduced and converted into purple formazan only by living cells. After the incubation period, the media containing MTT was removed, cells were washed in PBS, and 300 ml DMSO (Fisher Scientific, Pittsburgh, PA) was added to each well followed by incubation for 1 hour at room temperature, on an orbiting shaker protected from light. Samples were then transferred to 96-well plate and absorbance at 570 nm was determined using a Wallac 1420 Victor^3^V micro-plate reader (PerkinElmer, Shelton, CT). Each data point represents three (± S.D.) independent results; all data points were normalized to un-treated cells.

### eGFP gene targeting

Cells grown in complete medium supplemented with 10% FBS and experimental treatment where necessary were trypsinized and harvested by centrifugation. 2 × 10^6 ^cells were resuspended in 100 μl serum-free medium and transferred to a 4 mm gap cuvette (Fisher Scientific, Pittsburgh, PA). The oligonucleotide was added to a final concentration of 8 μM and the cells were electroporated (250 V, 13 ms, 2 pulses, 1s interval) using a BTX Electro Square Porator™ ECM 830 (BTX Instrument Division, Holliston, MA). The electroporated cells were then transferred to a 60 mm dish, recovered in complete medium supplemented with 10% FBS, and incubated at 37°C for 24 h prior to FACS analysis.

### Flow cytometry analysis

eGFP fluorescence was measured by a Becton Dickinson FACScalibur flow cytometer (Becton Dickinson, Franklin Lakes, NJ). Forty-Eight hours after electroporation, the cells were harvested and resuspended in FACS buffer (0.5% BSA, 2 mM EDTA, 2 μg/ml propidium iodide in PBS) and immediately processed. To analyze cell cycle, cells were seeded at a density of 1.5 × 10^6 ^in a 100 mm dish in complete medium supplemented with 10% FBS and the desired concentration of selenomethionine. After 24 h cells were trypsinized, resuspended in 300 μl cold PBS and fixed by addition of 700 μl cold 95% ethanol. Cells were incubated a 4°C for 16 hours, subsequently washed and resuspended in 500 μl of PBS containing 50 μg/ml propidium iodide and analyzed by FACScalibur for DNA content.

### Pulsed-field gel electrophoresis (PFGE)

Cells were seeded at a density of 1.5 × 10^6 ^in a 100 mm dish in complete medium supplemented with 10% FBS and selenomethionine at concentrations of 0, 100, 200, and 400 μM respectively, or MMS at 150 μM to serve as a positive control. After 24 h, the cells were trypsinized and 1 × 10^6 ^cells were melted in 1% low melt agarose (GIBCO, Invitrogen, Carlsbad, CA) in 50 mM EDTA. These agarose inserts were incubated in 50 mM EDTA, 1% N-laurosylsarcosine, 1 mg/ml proteinase K at 50°C, shaking, for 48 hours and subsequently washed four times in 1X TE buffer before loading onto a 1% pulsed field certified agarose gel (Bio-Rad, Hercules, CA). Samples were separated by electrophoresis for 24 h using a 120° field angle, 60 to 240s switch time, 4 V/cm (Bio-Rad, Hercules, CA), and visualized by ethidium bromide staining on an AlphaImager 2200 (Alpha Innotech Corp., San Leandro, CA).

### Western blot analyses

Cell extracts were prepared 24 h after the plasmids were introduced to the cells by electroporation. The cells were first washed with PBS and then suspended in lysis buffer (25 mM Tris, pH 7.5, 5 mM EDTA, 600 mM NaCl, 1 mM DTT, 0.1% NP40, 5 μg/ml leupeptin, 2 μM pepstatin, 1 mM PMSF, 10% glycerol) and incubated for 30 min followed by centrifugation at 15,000 × g for 30 min. The supernatant was analyzed for protein concentration using the BioRad Protein Assay. Proteins were separated on a 10% SDS-PAGE gel and electroblotted to a nitrocellulose membrane (BioRad, Richmond, CA). The membrane was incubated with α-p53 (DO-1), a mouse monoclonal antibody against total (wild-type and mutant) human p53 (Santa Cruz Inc., Santa Cruz, CA) at a 1:200 dilution. The secondary antibody, a bovine anti-mouse horse radish peroxidase conjugated-antibody (Santa Cruz Inc., Santa Cruz, CA) was used to visualize the primary reaction at a 1:5000 dilution, followed by the addition of Chemiluminescence ECL™ Reagents (Amersham, Piscataway, NJ) and hyperfilm (Kodak). Activated p53 was detected by α-p53 Ser^15 ^(PC386, Calbiochem, San Diego, CA) at a 1:1000 dilution, visualized by a goat α-rabbit secondary at a 1:2000 dilution. Ref-1 protein was detected using the Ref-1 (C-20): sc-334 affinity purified rabbit polyclonal antibody (Santa Cruz Inc., Santa Cruz, CA) at a 1:400 dilution; visualized by a goat α-rabbit secondary at a 1:2000 dilution. Actin expression was used as an internal control to normalize for protein levels; a goat β-actin antibody (Santa Cruz Inc., Santa Cruz, CA) in a 1:500 dilution, visualized by anti-goat IgG-HRP (Sigma, St. Louis, MI) with the Chemiluminescence ECL™ Reagents.

## Abbreviations

Selenomethionine (SeMet); Homologous recombination (HR); Pulsed-field gel electrophoresis (PFGE).

## Authors' contributions

TS designed and carried out all the experiments, experimental analyses and helped with manuscript preparation. EK conceived the study, data interpretation and drafted the manuscript. All authors read and approved the final manuscript.
